# Dispersive X-ray absorption spectroscopy using independent grazing-incidence focusing and convexly bent Bragg-crystal dispersing optics

**DOI:** 10.1107/S1600577525004953

**Published:** 2025-06-30

**Authors:** Juanjuan Huang, Adam P. Tornheim, Xianbo Shi, Mark Wolfman, Yanna Chen, Steve M. Heald, Shelly D. Kelly, George E. Sterbinsky

**Affiliations:** a X-ray Science Division Argonne National Laboratory Lemont IL60439 USA; b Chemical Sciences and Engineering Division Argonne National Laboratory Lemont IL60439 USA; c CLS@APS Canadian Light Source Saskatoon SK S7N 2V3 Canada; Bhabha Atomic Research Centre, India

**Keywords:** dispersive X-ray absorption spectroscopy, X-ray absorption fine structure

## Abstract

We present a modular instrument for dispersive X-ray absorption spectroscopy (DXAS) developed for the Advanced Spectroscopy Beamline at Sector 25 of the Advanced Photon Source. The setup employs a double-multilayer monochromator to provide X-rays with a broad energy bandwidth, Kirkpatrick–Baez mirrors for focusing, a convexly bent Bragg-crystal polychromator for energy dispersion, and a pixel-array detector to resolve all X-ray energies and collect their intensity simultaneously, thereby enabling acquisition of a full X-ray absorption spectrum in a single shot. The use of separate optics for X-ray focusing and energy dispersion provides high spatial resolution and avoids chromatic aberrations inherent in focusing bent-crystal optics, and a modular design makes implementation of the technique at other beamlines possible without requiring modifications to the upstream beamline configurations. Theoretical calculations are performed to determine optimal instrument operating parameters and demonstrate that an energy resolution better than the *K*-edge core-hole lifetime broadening can be maintained while providing a sufficient bandwidth for X-ray absorption near-edge structure spectroscopy through the full operating range of 5–11 keV. Additionally, instrument design, data analysis methods, and initial DXAS results on lithium–manganese–nickel oxide laminates are presented.

## Abstract 2

A dispersive X-ray absorption spectroscopy instrument has been developed and implemented at the Advanced Photon Source’s Advanced Spectroscopy Beamline. The instrument uses a convexly bent Bragg-crystal analyzer to disperse a polychromatic X-ray beam and independent Kirkpatrick–Baez mirrors for focusing, thereby providing a simple and flexible method for acquisition of X-ray absorption spectra, with the absorption at each energy measured simultaneously in a single shot.

## 1. Introduction

X-ray absorption fine-structure (XAFS) spectroscopy, which often refers to X-ray absorption spectroscopy (XAS) in the hard X-ray range, is a prime characterization technique for studying chemical speciation and structural properties of both crystalline and amorphous matter. One XAFS spectrum is categorized into two regimes: XANES (X-ray absorption near-edge structure) (Henderson *et al.*, 2014 ▸) and EXAFS (extended X-ray absorption fine-structure) (Lee *et al.*, 1981 ▸), depending on the difference between the energies of the absorbed X-rays and the threshold energy of the core level excitation. This technique offers insights into both the electronic and geometric structures of materials. More specifically, XAFS provides information on oxidation states (Kelly *et al.*, 2008 ▸; Hona *et al.*, 2021 ▸; Wilke, 2024 ▸), bond lengths (Mikkelsen & Boyce, 1982 ▸; Kelly *et al.*, 2002 ▸; Sterbinsky *et al.*, 2012 ▸), coordination numbers (Ravel & Kelly, 2007 ▸) and local symmetry (Westre *et al.*, 1997 ▸; Vedrinskii *et al.*, 1998 ▸), which has led to its application in a wide range of research fields.

The conventional XAS acquisition scheme relies on step-scanning a double-crystal monochromator (DCM) through a range of Bragg angles, thus varying the outgoing monochromatic X-ray energy. The X-ray absorption of a sample placed downstream of the monochromator is measured at each sequential energy step over a specified energy range in order to obtain a complete spectrum. Measurement of an entire XAS spectrum by step-scanning a DCM typically requires a few minutes. Acquisition times can be reduced by continuously scanning or oscillating a DCM at high speed during data acquisition, which is referred to as quick-XAFS (QXAFS) (Fonda *et al.*, 2012 ▸). Monochromators operated in continuous scanning mode can acquire full XAFS spectra in seconds while oscillating DCMs are able to achieve millisecond-scale acquisition (Frahm, 1988 ▸, 1989 ▸; Richwin *et al.*, 2001 ▸; Frahm *et al.*, 2010 ▸; Fonda *et al.*, 2012 ▸; Müller *et al.*, 2016 ▸; Rosa *et al.*, 2024 ▸).

Another scheme to measure XAS uses a polychromator to disperse incoming X-rays and form an energy gradient through a continuous variation of Bragg angles along the polychromator’s curved surface (Pascarelli *et al.*, 2016 ▸). The polychromator is typically concavely bent in order to focus the polychromatic X-ray beam onto a sample. A position-sensitive detector placed after the sample resolves the spatially correlated energies and generates XAS spectra after signal processing. This method is typically referred to as dispersive XAS (DXAS). Compared with conventional XAS, DXAS eliminates the need to scan the monochromator and captures an entire spectrum in a single data acquisition event, or, colloquially, in a single shot.

Since its initial development in the 1980s (Kaminaga *et al.*, 1981 ▸; Matsushita & Phizackerley, 1981 ▸; Phizackerley *et al.*, 1983 ▸), the DXAS technique has proliferated to synchrotrons throughout the world (Pascarelli & Mathon, 2017 ▸). Recent DXAS synchrotron beamlines around the world include the Energy-Dispersive X-ray Absorption Spectroscopy (ED-XAS) beamline at the European Synchrotron Radiation Facility (Sévelin-Radiguet *et al.*, 2022 ▸), beamlines BL14B1 (Okajima *et al.*, 2007 ▸) and BL28B2 (Kato *et al.*, 2016 ▸) at SPring-8, the Energy Dispersive EXAFS (EDE) beamline at Diamond Light Source (Diaz-Moreno *et al.*, 2018 ▸), the Optique Dispersive EXAFS (ODE) beamline at SOLEIL (Baudelet *et al.*, 2011 ▸), Dynamic line (D-line) at Shanghai Synchrotron Radiation Facility (He *et al.*, 2022 ▸), the dispersive X-ray absorption spectroscopy beamline at Brazilian Synchrotron Light Laboratory (Cezar *et al.*, 2010 ▸), the time-resolved XAS (TRXAS) beamline at Synchrotron Light Research Institute (Limphirat *et al.*, 2020 ▸), BL-08 at the Indus-2 synchrotron (Patra *et al.*, 2019 ▸), beamline NW2A of the Photon Factory Advanced Ring (Shirasawa *et al.*, 2024 ▸), and beamline BL-5 of the Synchrotron Radiation Center of Ritsumeikan University (Katayama *et al.*, 2015 ▸). In addition to these dedicated DXAS beamlines, there are multipurpose beamlines equipped with DXAS capabilities, such as the Bundesanstalt für Materialforschung und -prüfung (BAMline) at BESSY-II (Buzanich *et al.*, 2016 ▸) and the Dynamic Compression Sector (DCS) at the Advanced Photon Source (Das *et al.*, 2020 ▸).

As a single-shot technique, DXAS offers significant advantages for time-resolved studies. Consequently, it has been widely applied in X-ray free-electron laser (XFEL) facilities, including the Linac Coherent Light Source (LCLS) (Harmand *et al.*, 2023 ▸), the SPring-8 Angstrom Compact free-electron LAser (SACLA) (Inubushi *et al.*, 2021 ▸), the European XFEL (Khakhulin *et al.*, 2020 ▸), FLASH (Free Electron Laser in Hamburg) (Brenner *et al.*, 2019 ▸), and the Swiss Free Electron Laser (Juranić *et al.*, 2024 ▸). A comprehensive review of spectroscopy techniques at XFEL facilities is provided by Bergmann *et al.* (2021 ▸).

In addition, recent years have seen significant progress in the development of laboratory XAS. Due to its fast and motion-free acquisition scheme, dispersive XAS has gained popularity in various laboratory configurations, as summarized in recent review articles (Zimmermann *et al.*, 2020 ▸; Malzer *et al.*, 2021 ▸).

In this paper, we present a dispersive XANES instrument at the Advanced Spectroscopy Beamline (ASB) of the Advanced Photon Source (APS). A polychromatic X-ray source is provided by either of two Mo/B_4_C double multilayer monochromators (DMMs), each having a bandwidth over 1%. This intense polychromatic spectrum is ideal for DXAS. A pair of Kirkpatrick–Baez (KB) mirrors focus the polychromatic beam on the sample. As an energy-dispersive optic, we employ a convexly bent Bragg diffracting crystal downstream of the sample.

The setup is simple to implement and does not require additional adaptations of the beamline optical layout, thereby efficiently expanding the capabilities of the beamline. Compared with most DXAS setups implemented at synchrotrons worldwide, which use concave crystals as polychromators serving both as dispersive and focusing elements, our setup separates the optical elements that provide X-ray focusing and energy dispersion into independent components. This opens the possibility for nano-focusing by employing low-figure-error KB mirrors (Mimura *et al.*, 2010 ▸; Matsuyama *et al.*, 2017 ▸), which has not been possible using dispersive bent Bragg or Laue polychromators. Additionally, using independent grazing-incidence optics for focusing significantly simplifies the design of the dispersive polychromator, relaxes the requirements for polychromator bending accuracy, and avoids the chromatic aberrations inherent in X-ray focusing with bent crystals, which can cause artifacts in spectra acquired from micro-scale inhomogeneous samples (Sanchez *et al.*, 2017 ▸). The use of a convexly bent crystal also increases the angular dispersion of the polychromator relative to a flat or concave crystal, which improves the energy resolution of a pixel-array detector placed a given distance away.

Placing the crystal polychromator downstream of the sample increases the flux on the sample and thereby raises the need for effective radiation damage management, such as the design of compact cryogenic systems (Yoneyama *et al.*, 2021 ▸). However, the placement of the polychromator also results in a reduction of the X-ray flux that it receives compared with designs where it is positioned further upstream. Consequently, cooling requirements for the polychromator are more relaxed compared with front-end optics, making polychromator manipulation considerably more practical and enabling transferability to different beamline endstations.

The ability of this instrument to collect an X-ray absorption spectrum in a single shot enables fast XANES acquisition at the ASB, in principle reaching the millisecond range, in contrast to the current Si(111) DCM, which is not of the oscillating type. This improvement is particularly beneficial for studying dynamic reactions or performing fast XANES mapping when combined with fly scanning of the sample stage. In particular, the instrument could be used to examine the kinetics of chemical processes *in situ* or *in operando* or to study physical phenomena on nanosecond timescales using pump–probe techniques (Guzelturk *et al.*, 2024 ▸; Diroll *et al.*, 2023 ▸; Liu *et al.*, 2020 ▸).

## 2. The Advanced Spectroscopy Beamline and DXAS instrument

The DXAS instrument is implemented within the 25-ID-C endstation of the ASB, which is a branch of the canted undulator beamline at Sector 25 of the APS, Argonne National Laboratory. As a successor to the multipurpose beamline 20-ID (Heald *et al.*, 2007 ▸), it offers spectroscopy techniques including X-ray absorption spectroscopy (Wan *et al.*, 2021 ▸), X-ray emission spectroscopy (Solovyev *et al.*, 2021 ▸), high energy resolution fluorescence detected (HERFD) XAS (Chen, Finfrock *et al.*, 2021 ▸), and micrometre-scale imaging techniques including confocal X-ray imaging (Choudhury *et al.*, 2015 ▸) and X-ray fluorescence (XRF) mapping (Kachenko *et al.*, 2010 ▸). Beyond these characterization methods, new opportunities for techniques requiring a wider bandpass are made possible by the newly designed ASB monochromator, which can operate with a Si (111) double-crystal set or either of two Mo/B_4_C double-multilayer sets. The two DMMs have different bilayer periods and energy bandwidths of ∼1% and ∼3% for the shorter- and longer-period Mo/B_4_C pairs, respectively, which increase the outgoing X-ray flux by over one order of magnitude compared with the Si (111) DCM.

### 2.1. Source, optics, and instrumentation

The source of the ASB changed in 2024, with the completion of an upgrade of the APS to a fouth-generation synchrotron radiation facility. The upgraded APS (APS-U) electron beam has a substantially reduced horizontal emittance compared with that of the pre-upgrade APS electron beam, and it operates with an increased storage ring current [see chapter 2 of the APS-U Final Design Review Report (https://www.aps.anl.gov/APS-Upgrade/Documents)]. The new source characteristics, shown in Table 1 ▸, result in the X-rays that it produces having increased coherence and flux. Note that the experimental results presented in this manuscript were measured before the APS upgrade.

**Figure 1:**
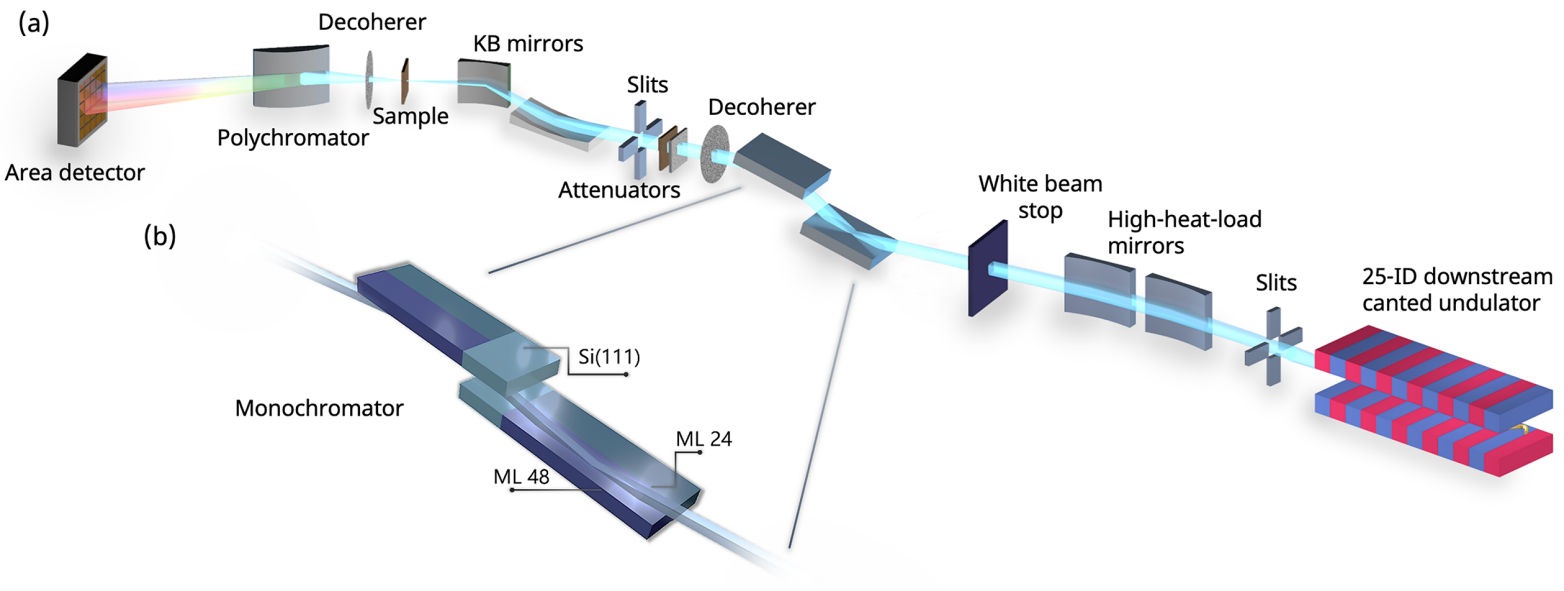
(*a*) Schematic of the DXAS instrument and ASB at APS Sector 25. (*b*) Simplified schematic of the ASB monochromator.

At Sector 25, X-rays are generated from the electron beam by two canted undulators. Table 2 ▸ gives the undulator specifications. The downstream canted undulator provides the source for the ASB, a schematic of which is shown in Fig. 1 ▸(*a*). The X-rays produced by the downstream canted undulator reflect from a pair of horizontally deflecting high-heat-load mirrors, which increase the separation between the beams generated by the two canted undulators. The mirrors are made from silicon and have three different vertically aligned deflecting regions. These are uncoated silicon, rhodium-coated, and platinum-coated regions, which result in respective cutoff energies of 12.6, 23.2, and 33.7 keV when operating with the required 2.5 mrad angle of incidence. The downstream mirror can be bent to collimate or focus the outgoing X-ray beam. A white-beam stop intercepts any unreflected X-rays, while the reflected pink beam proceeds to the monochromator.

The ASB monochromator contains three distinct pairs of optics, as shown in Fig. 1 ▸(*b*): Si(111) crystals and sputter-deposited Mo/B_4_C multilayers (MLs) with bilayer thickness of 24 Å (ML 24) and 48 Å (ML 48) (Conley Jr *et al.*, 2014 ▸). Their specifications are detailed in Table 3 ▸. The monochromator is switched to a multilayer set when operating the DXAS instrument, as it requires a polychromatic incident spectrum. The range of Bragg angles for both multilayer sets is 0.7–1.5°, corresponding to operating energy ranges of 4.9–10.7 keV and 9.9–21.5 keV for ML 48 and ML 24, respectively. The bandwidths of the multilayer pairs can be determined by simulating the optics’ reflectivity. Simulations were conducted using the Python package *XOPPYLIB* (https://github.com/oasys-kit/xoppylib) and the specifications in Table 3 ▸. Similar calculations can also be performed through graphical user interfaces such as *XOP* (Sanchez del Rio & Dejus, 2011 ▸), *OASYS* (Rebuffi & Sanchez del Rio, 2017 ▸), or *XRT* (Klementiev & Chernikov, 2014 ▸).

**Figure 2:**
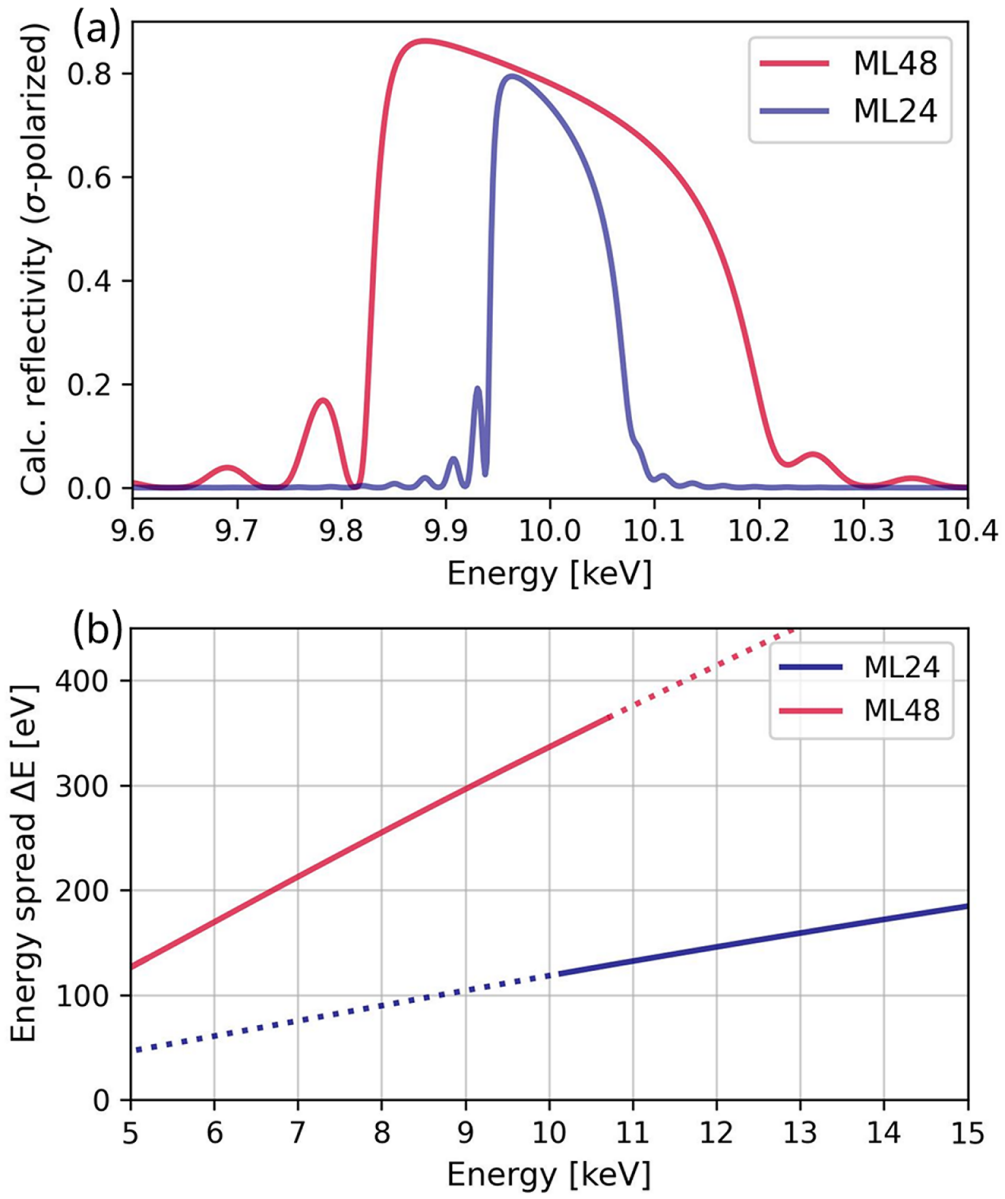
(*a*) Simulated reflectivity (σ-polarized) for the two multilayer sets with X-ray energy centered at 10 keV. The calculated FWHMs are 337 eV and 118 eV for ML 48 and ML 24, respectively. (*b*) The FWHM of the reflectivity profiles for ML 48 and ML 24 simulated over the range of 5–15 keV. The solid lines represent the operating energy ranges of the multilayers at APS Sector 25. The operational ranges are determined by the geometric design constraints of the DMM, whereby the second multilayer is not positioned to intercept X-rays of energies outside of the operating range that are reflected from the first multilayer.

**Figure 3:**
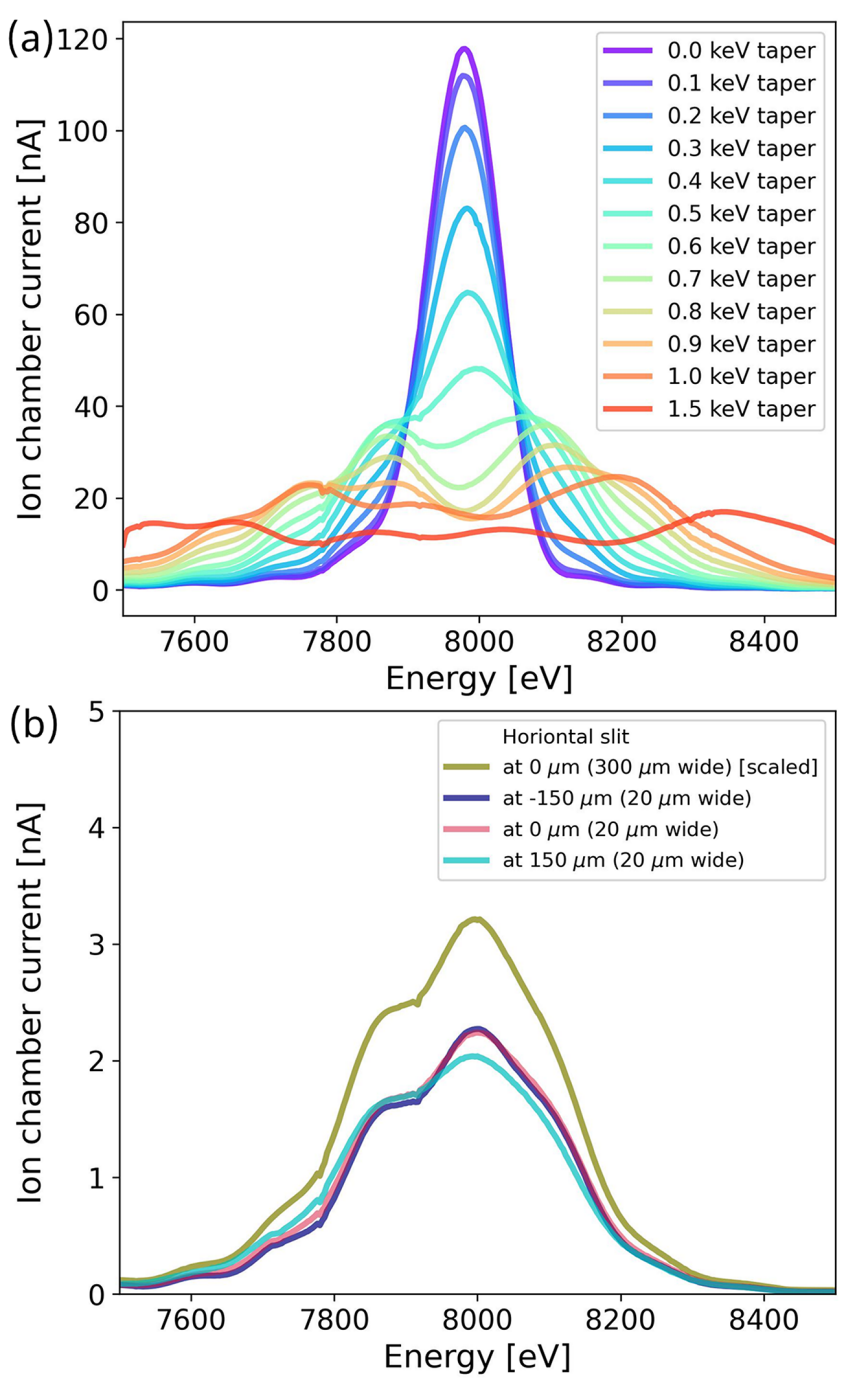
(*a*) APS-U undulator (U28) spectra through 300 µm × 300 µm slits in front of the KB mirrors measured at different undulator tapering values by scanning the Si(111) monochromator and recording ion chamber readings downstream of the slits. (*b*) U28 spectra measured through 20 µm × 300 µm (horizontal × vertical) slits at different horizontal positions (−150 µm, 0 µm, 150 µm) with 0.5 keV undulator tapering and compared with the spectrum measured through 300 µm × 300 µm slits (downscaled by a factor of 15). The reduced intensity at 150 µm is attributed to a drop in the storage ring current. These scans confirm that X-ray energies are homogeneously distributed within the 300 µm × 300 µm slits.

Reflectivity simulations were conducted for X-ray energies between 5 and 15 keV, and the corresponding energy spread is plotted in Fig. 2 ▸(*b*). For a given energy, the energy spread is taken as the full width at half-maximum (FWHM) value of the reflectivity curve, as shown in Fig. 2 ▸(*a*) for both ML 48 and ML 24 at 10 keV. The resulting FWHM values of these two reflectivity curves are 337 eV for ML 48 and 118 eV for ML 24. The available energy range of the DXAS spectrometer is determined by the bandwidth of the multilayer monochromator, which is ∼3% in the primary operating range of ML 48. This bandwidth roughly matches that of APS undulator A (U33). The bandwidth of the APS-U undulator (U28) is reduced with respect to that of U33, and to match the bandwidth of ML 48 its magnets can be tapered. This is illustrated in Fig. 3 ▸(*a*), which shows the spectra of U28 at a nominal energy of 8 keV and different tapering values measured through a 300 µm × 300 µm slit by scanning the Si(111) monochromator and recording the current in an He-filled ion chamber. The FWHM of the spectra increases with increasing tapering. To ensure that the FWHM of the undulator is at least 3% when using ML 48, the undulator can be tapered by 0.5 keV, which results in an FWHM of 330 eV, corresponding to a 4% bandwidth.

The energy ranges imposed by the bandwidths of the multilayers are sufficient for the measurement of XANES spectra but limit the ability to collect EXAFS. To illustrate, ML 48’s bandwidth corresponds to a *k*-range of 6.3 Å^−1^ at 5 keV and 9.4 Å^−1^ at 10 keV, as calculated using *k* = 0.512(*E* − *E*_edge_)^1/2^. For this reason, the DXAS instrument will primarily be utilized in the XANES domain.

The X-ray beam from the ML monochromator enters the experimental endstation, where the DXAS instrument is deployed. The first component is a decoherer, which is created by attaching six sheets of copy paper to a shaft that is continuously rotated by a motor at ∼200 Hz. Paper is a strong scatterer, as it is composed of fibers with a multi-scale density variation spanning from nanometres to hundreds of micrometres (Bech *et al.*, 2010 ▸). It acts as a random phase plate that effectively reduces the spatial coherence of the X-ray beam and removes X-ray beam structures caused by phase contrast (Cloetens *et al.*, 1996 ▸). Graphite foil has also been employed to decohere X-rays (De Andrade *et al.*, 2011 ▸). The decoherer is crucial for flatfield correction, as variations in the beam structure could introduce significant artifacts.

While the decoherer was found to be effective for the experiments described herein, more coherent X-ray sources, such as those at fourth-generation synchrotrons, may increase beam structure variation. If these variations increase to an extent that they can no longer be corrected solely by the decoherer, additional post-processing methods can be applied. In one such method, principal component analysis is applied to a set of flat fields collected at different times to extract the most significant variations, forming eigen flat fields that can then be used for normalization. This method significantly reduces the errors caused by beam instability at synchrotrons and XFELs (Nieuwenhove *et al.*, 2015 ▸).

The decoherer is followed by a set of Al and Mo attenuators that can be inserted into the beam path to control the incident flux on the sample and downstream instrument components. For the proof-of-principle experiments described herein, the beam was passed through Al or Mo attenuators, depending on X-ray energy, to reduce beam-induced damage of Kapton windows at various locations and decrease the generation of ozone in the experimental hutch by the high intensity X-rays. In the future, full enclosure of the DXAS instrument and beam path in a helium purged environment will eliminate these concerns.

After being homogenized and potentially attenuated, the beam is reduced to a size of about 180 µm × 300 µm (horizontal × vertical) by a set of slits to align with the maximum acceptance of a set of KB mirrors (Kirkpatrick & Baez, 1948 ▸), which focus the X-ray beam. A smaller horizontal acceptance is used to compensate for the large horizontal source size of the APS prior to the upgrade and provide a focal spot size similar to that obtained in the vertical direction. With the reduction in horizontal source size provided by the APS-U, a mirror with larger acceptance can achieve an equivalent focus. Therefore, with the APS-U source, a larger slit size of 300 µm × 300 µm will be used to match the increased acceptance of a longer KB mirror, as detailed in Table 2 ▸. The achromatic nature of the KB mirrors eliminates the need for mirror realignment when changing X-ray energies. The mirrors are profile-coated to provide an elliptical shape in the meridional direction and have a rhodium reflecting surface (Liu *et al.*, 2003 ▸). Optimal focus is obtained at an incident angle of 3.0–3.2 mrad for both the horizontal and the vertical mirrors, which provides a cutoff energy of 20.6–21.9 keV. The focal distance is 90 mm from the center of the downstream vertical mirror.

**Figure 4:**
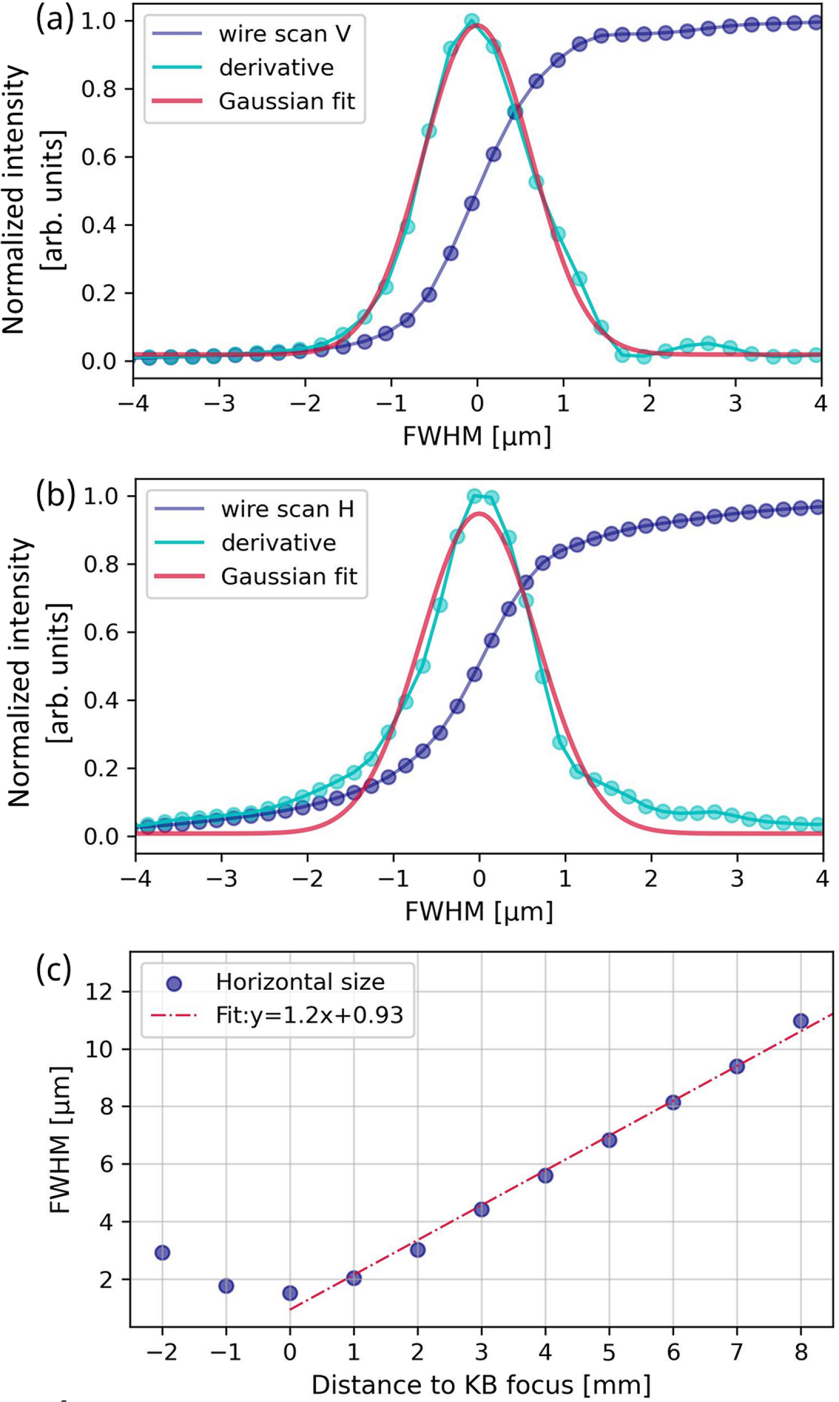
Wire scans (blue dots) through the X-ray beam at the focal spot of the KB mirrors along the (*a*) vertical direction and (*b*) horizontal direction. The derivatives of the scans are shown by cyan dots and are fitted by Gaussian functions (red lines). The derived FWHM from the Gaussian fits are 1.5 µm and 1.6 µm for the vertical and horizontal directions, respectively. (*c*) Horizontal spot size measured as a function of X-ray propagation distance (blue dots). The slope of the linear fit (red dashed line) gives the horizontal divergence, *i.e.* 1.2 mrad.

The focused beam size was measured by scanning a platinum wire with a diameter of 50 µm across the focused beam. Fig. 4 ▸ displays both vertical and horizontal scans of the wire, which reveal a focused beam size of 1.5 µm × 1.6 µm (V × H) FWHM at the sample position. This focal size impacts the spectrometer’s energy resolution and determines the spatial resolution for scanning probe imaging.

The optimal optical configuration of the DXAS instrument depends upon the horizontal divergence of X-rays after passing through the sample, as discussed in Section 3 below. To determine the divergence, we measured the horizontal spot size at various X-ray propagating distances. The resulting horizontal divergence is 1.2 mrad for X-rays exiting from the KB mirrors, as determined by the linear fit presented in Fig. 4 ▸(*c*). The sample is placed at the KB focal spot, which is about 54 mm away from the downstream face of the KB mirror enclosure. This gives sufficient space for mounting compact *in situ/operando* sample cells.

**Figure 5:**
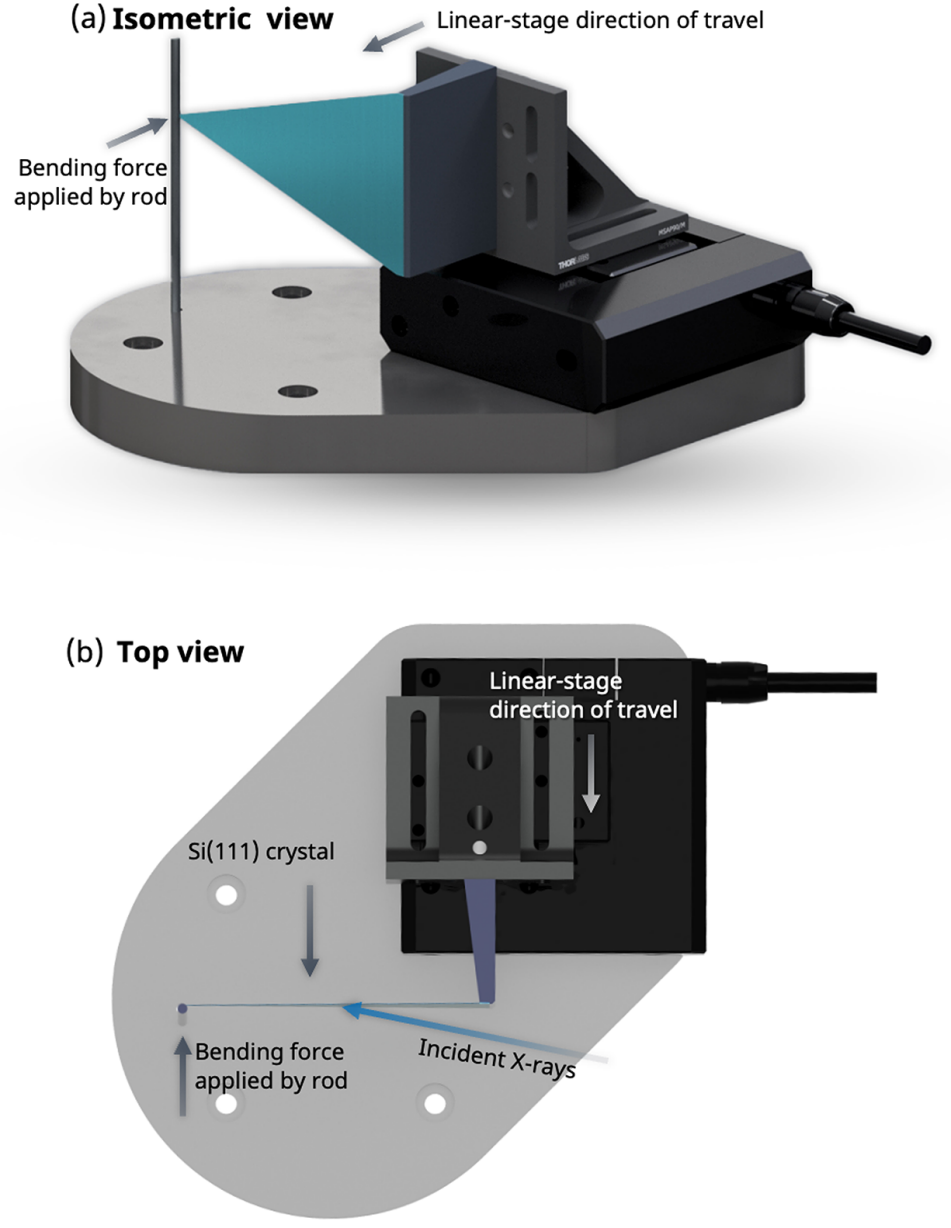
(*a*) Isometric and (*b*) top views of the polychromator design drawing. The Si (111) crystal is shown in blue-green.

**Figure 6:**
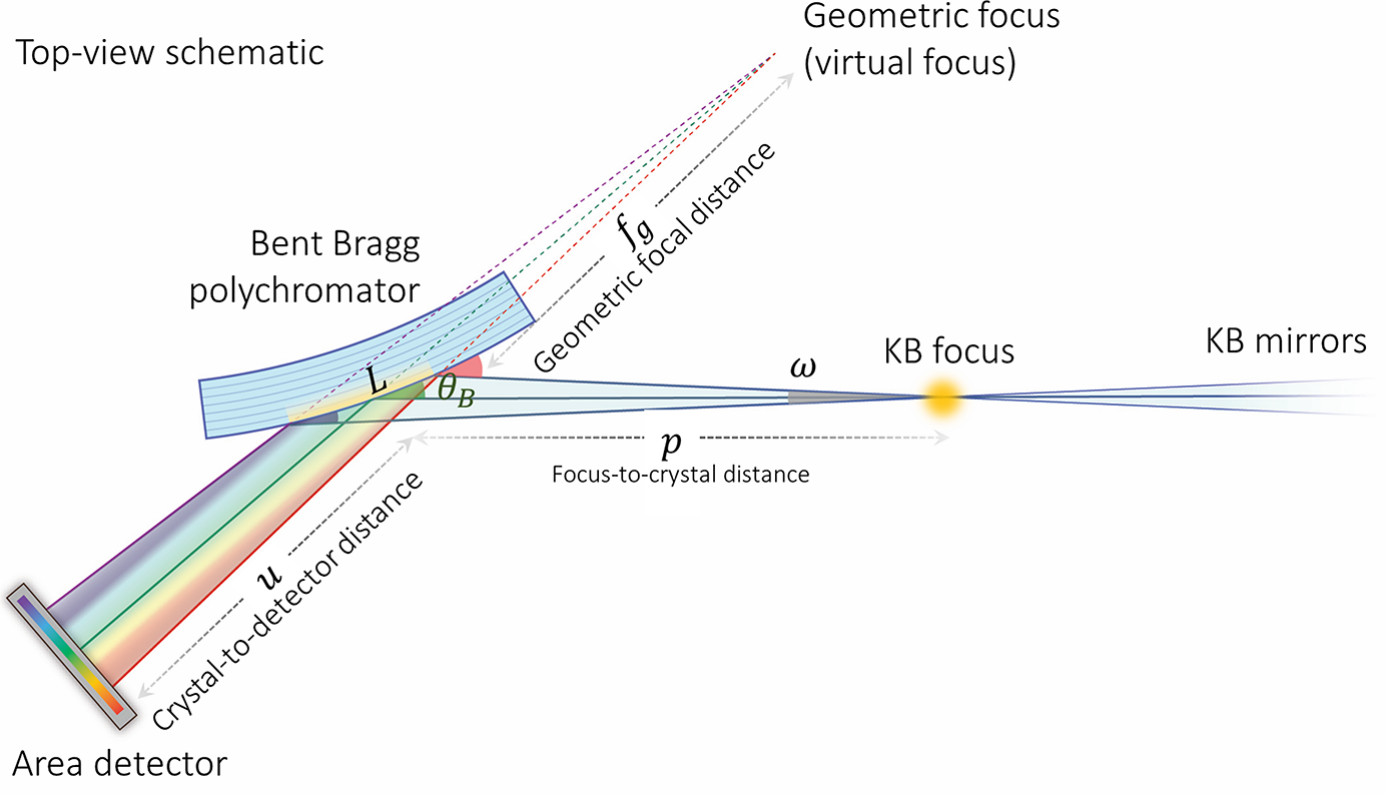
Top-view schematic of the DXAS instrument.

The sample is followed by the Bragg polychromator, the design of which is shown in Fig. 5 ▸. Silicon wafers were cut into triangles with a base length of 38.1 mm and two equivalent side lengths of 63.5 mm. The triangular-shaped crystal with uniform thickness will bend to a circle with a constant radius (Henins, 1987 ▸). The base of the crystal is fixed to a 3D-printed wedge by adhesive ep­oxy. The wedge is mounted to a motorized stage (M-110.12S model, PI GmbH & Co. KG, Germany) via an L-bracket. The crystal bending is tuned by driving the stage while keeping the crystal tip pressed against a vertical rod. The bending force is applied to the crystal tip, similar to schemes used for X-ray monochromators (Lemonnier *et al.*, 1978 ▸). The polychromator assembly is further mounted on top of a rotation stage, a vertical lift stage, and two horizontal linear stages, enabling adjustment of θ, *x*, *y*, and *z* in order to accommodate various energy ranges and facilitate alignment of the optic. The divergent X-rays, in combination with the convexly bent crystal, result in a continuous change of Bragg angles along the crystal surface, illustrated in Fig. 6 ▸. This leads to a spatially resolved energy dispersion of the polychromatic X-rays.

An energy–spatial correlation is also present in the undulator spectrum, and, due to the greatly reduced horizontal emittance of the APS-U electron beam, this correlation is more pronounced in the horizontal direction of X-rays produced by the APS-U. Because diffraction of X-rays with different energies from the crystal analyzer is inhomogeneous, with each energy being diffracted from a different region, the uniformity of the undulator source must also be ensured. Fig. 3 ▸(*b*) shows undulator spectra with the slit size further reduced to 20 µm × 300 µm (horizontal × vertical). These undulator spectra were collected with the slit at different horizontal positions, specifically at the center (0 µm), left edge (−150 µm), and right edge (150 µm). These spectra were then compared with the scaled spectrum measured at the center using a 300 µm × 300 µm slit. All spectral shapes resemble each other, confirming that X-ray energies are homogeneously distributed within the 300 µm × 300 µm aperture. Therefore, the increased energy–position correlation of the APS-U will not reduce the energy range diffracted from the polychromator.

The X-rays diffracted from the polychromator are counted by a pixel-array detector placed at the 2θ_B_ angle. Due to the energy dispersion created by the polychromator, each horizontal column of pixels detects the intensity of X-rays having a different energy, effectively measuring the X-ray absorption spectrum. The detector used for the DXAS instrument is a photon-counting pixel-array detector (LAMBDA 250K, X-Spectrum GmbH, Germany). It exhibits high X-ray quantum efficiency within our Bragg polychromator energy regime (<10 keV). Photon-counting detectors offer several advantages, including noise-free image acquisitions, sharp point spread functions, a wide dynamic range, and high frame rates (Förster *et al.*, 2019 ▸). Dual thresholds were used for measurements presented herein, with a low threshold of 3 keV and a high threshold of 15 keV. Additional detector specifications, as well as those of various other instrument components, are summarized in Table 2 ▸.

### 2.2. Flux and power

**Figure 7:**
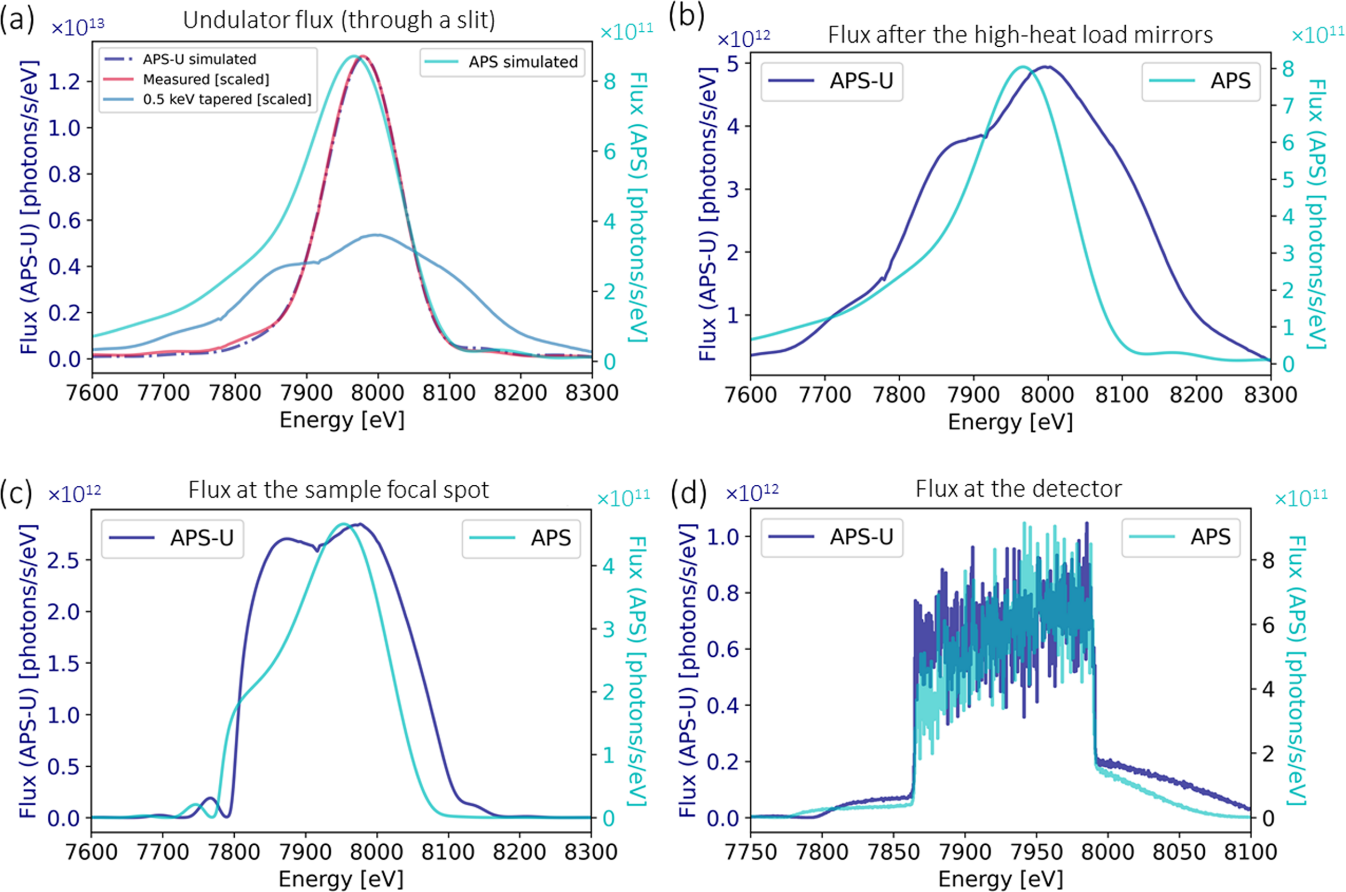
Flux simulation using the *XOP* and *SHADOW* modules in the *OASYS* software package. (*a*) Simulated X-ray flux of APS and APS-U undulators with white beam slits matching the acceptance of the KB mirrors. For APS-U, we scaled the spectrum measured without undulator tapering to the simulation and then applied the same scale factor to the spectrum measured with 0.5 keV tapering. (*b*) Expected flux after the high-heat-load mirrors, considering the silicon stripes. (*c*) Expected flux at the sample focal spot, accounting for the reflectivity of the ML 48 monochromator and Rh-coated KB mirrors. (*d*) Expected flux at the detector. Note that the simulation does not account for any additional losses or experimental inefficiencies.

The flux and power incident on various optical components are important considerations in the design of beamline instrumentation, which dictate potential for radiation damage and need for cooling. We have therefore calculated the flux and power of X-rays produced by the beamline, the spectral shape of which can vary with the amount of undulator tapering, multilayer angle, and both alignment and spectral match between optical components. The flux and power output from several beamline and instrument components at the representative energy of 8 keV are given in Table 4 ▸, and the corresponding spectra are shown in Fig. 7 ▸. Calculations were carried out using *XOP* (Sanchez del Rio & Dejus, 2011 ▸) and *SHADOW* (Sanchez del Rio *et al.*, 2011 ▸; Rebuffi & Sanchez del Rio, 2016 ▸), which are also part of the *OASYS* ray tracing package (Rebuffi & Sanchez del Rio, 2017 ▸). For APS-U, the spectrum measured without undulator tapering was scaled to the simulation and the same scale factor was then applied to the spectrum measured with 0.5 keV tapering. The total flux is determined from the integral of the undulator spectrum, and Table 4 ▸ shows the flux of the undulator that can be accepted by the DXAS instrument, which is limited by the lengths of the KB mirrors. A large portion of the power is reduced by narrowing the water-cooled white beam slits to match the acceptance size of the KB mirrors, as shown in Table 5 ▸. Seventy to eighty percent of the X-ray beam power is removed by the high-heat-load mirrors and ML 48, which are water- and liquid nitro­gen-cooled, respectively. The remaining power can be accepted by the KB mirrors and subsequent polychromator without the need for cooling, though enclosure in helium environments is desirable to mitigate X-ray induced deposition of surface carbon.

The flux will increase by about an order of magnitude after the upgrade of the APS, as shown in Table 4 ▸. Several factors contribute to this increase, including the reduced electron beam emittance, doubled electron beam current, increased acceptance of the horizontal KB mirror, and collimation of the beam using the downstream high-heat-load mirror.

## 3. Spectrometer properties

### 3.1. Bandwidth

The divergence of the X-rays incident on the crystal and the crystal’s curvature result in a horizontal energy gradient. The energy bandwidth of the spectrometer is determined by the range of diffracted angles. The total angular spread (Δθ) can be calculated by adding the horizontal X-ray divergence, ω, to the central angle of the crystal’s arc,
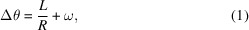
where *R* is the meridional bending radius of the crystal. The arc length of the crystal’s surface illuminated by X-rays is denoted by *L* and can be estimated by
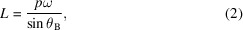
where *p* is the distance from the KB focal spot to the crystal and θ_B_ is the Bragg angle. This equation is valid when both *R* and *p* are much greater than *L*, and the crystal is assumed to be sufficiently long to capture the full incident diverging beam. Combining equations (1) and (2) yields
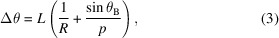
which gives the Bragg angle range in terms of the of crystal analyzer properties and position. Focusing, or concave, analyzer crystals are much more frequently employed for DXAS. In that case, the plus sign is replaced by a minus sign in equations (1) and (3), which illustrates that the use of a convex crystal provides an advantageous increase in the Bragg angle distribution.

For small Δθ, the crystal’s total energy spread, Δ*E*, can be calculated from the angular spread using the derivative form of Bragg’s law, 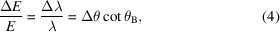
where *E* and λ represent the X-ray energy and wavelength, respectively. By combining equations (2), (3) and (4), the total energy spread created by the dispersive optic is
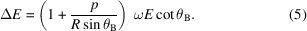
Equation (5) expresses the energy spread independent of the illuminated analyzer arc length and as a function of only readily known static beamline and analyzer properties. It can, therefore, be used to readily determine instrument energy bandwidth for a given set of operating parameters.

### 3.2. Energy resolution

The energy resolution of the DXAS instrument is determined by the monochromatic angular spread (denoted as δθ_res_), which has contributions from multiple factors (Pascarelli & Mathon, 2017 ▸). These include the X-ray source size (δθ_ss_), which is the KB focal spot size in this case, the spatial resolution of the pixel-array detector (δθ_det_), and the intrinsic energy resolution of the analyzer crystal (δθ_int_). Their relationship is expressed as
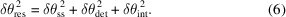
The total energy resolution can be calculated from δθ_res_ using equation (4), which gives
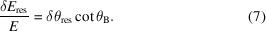
In order to determine the total energy resolution of the instrument, contributions from the detector, source, and crystal can be calculated, as discussed below.

#### 3.2.1. Spatial resolution of the pixel-array detector

**Figure 8:**
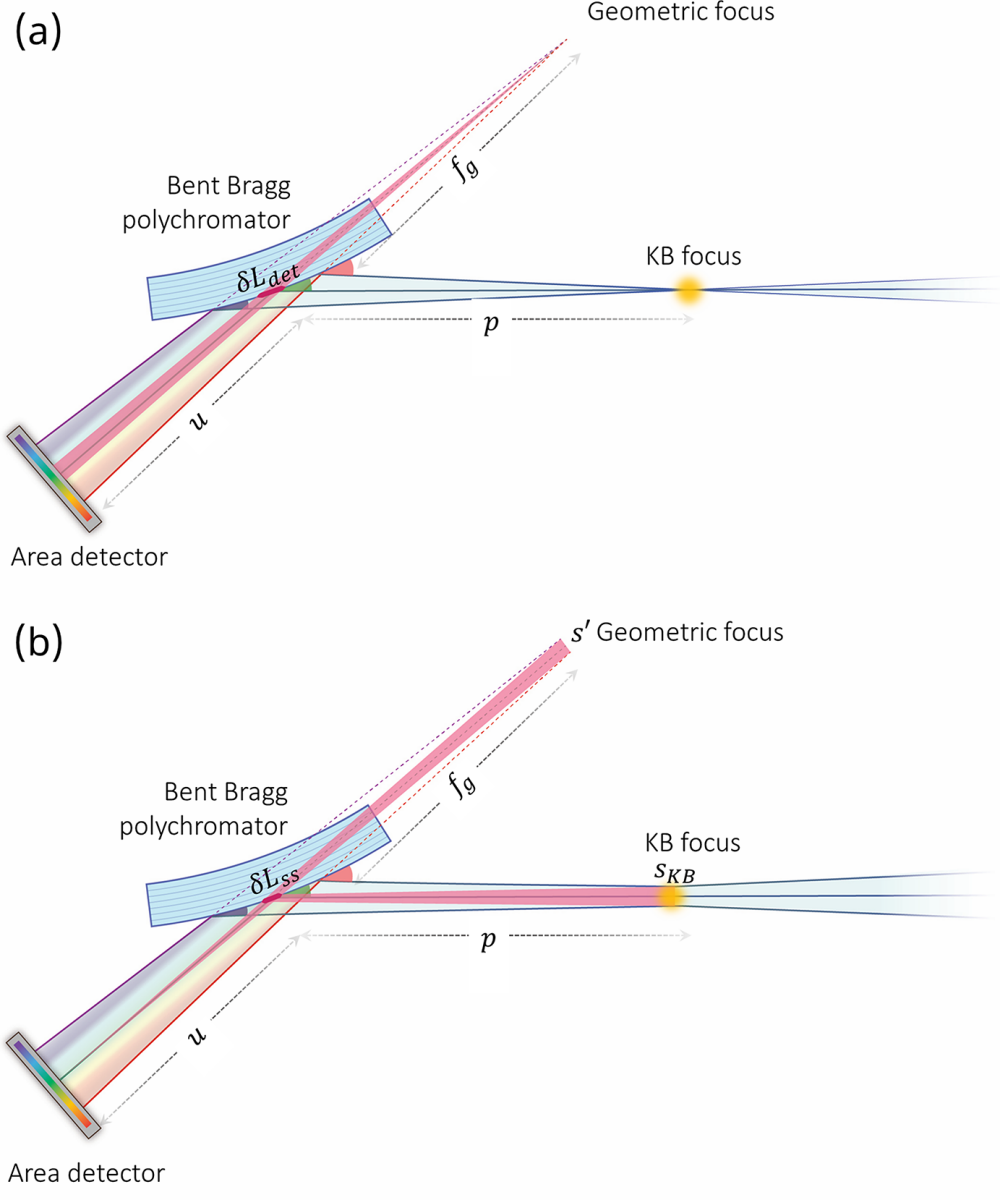
Top-view schematics of the DXAS instrument illustrating the energy broadening effects induced by (*a*) the finite pixel size of the pixel-array detector and (*b*) the finite source size, *e.g.* the KB focus size.

Fig. 8 ▸(*a*) shows the angle of view of a pixel from the polychromatic focal point in order to illustrate the broadening of the energy resolution by a pixel of finite size. From the perspective of the detector, the point in space from which the X-rays appear to emanate in the absence of the crystal is the virtual geometric focus (*f*_g_), which is related to the analyzer Bragg angle by Coddington’s equation for meridional focusing (Willmott, 2019 ▸) adapted for a convex optic,
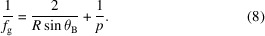
The geometric focal point and finite-sized pixel define a triangle [shaded red in Fig. 8 ▸(*a*)], and when this triangle is projected onto the crystal it defines a region δ*L*_det_ shown by the red line on the crystal surface in Fig. 8 ▸(*a*) and given by
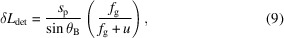
where *u* is the distance from the analyzer crystal to the detector and *s*_p_ is the width of a detector pixel. The region δ*L*_det_ is correlated to a Bragg angle range that defines the pixel resolution, which from equation (3) is found to be
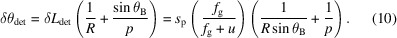
This equation is similar to that for a concave crystal (Tolentino *et al.*, 1988 ▸), but here the resolution scales as the inverse of the distance from the detector to the virtual focus of the convex crystal rather than the distance from the detector to the true focus of a concave crystal.

#### 3.2.2. Focal spot size of the KB mirrors

Fig. 8 ▸(*b*) shows the distribution of X-ray angles striking the detector at a single point when the source has a finite size. The rays striking at different angles have different energies and thus degrade the energy resolution of the instrument. When the source size is finite, the virtual focus will also have a finite size (*s*′). In this case, the source size is that of the KB mirror focal spot (*s*_KB_), and it is related to the size of the virtual focus by
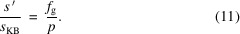
Similar to the finite pixel and virtual point source discussed in the preceding section, the finite virtual source and a point on the detector form a triangle [shaded red in Fig. 8 ▸(*b*)]. When this triangle is projected onto the crystal, it defines a region δ*L*_ss_ indicated by the red line on the crystal surface in Fig. 8 ▸(*b*) and related to the virtual source size by
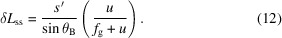
The range of Bragg angles correlated with the region δ*L*_ss_ is given by
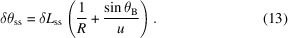
This is similar to equations (3) and (10), with δ*L*_ss_/*R* contributed by the crystal curvature and 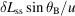 contributed by the converging angular spread of rays originating from different points along the finite source. By combining equations (11), (12), and (13), one obtains the angular resolution in terms of the source size, which is
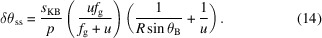
By using equation (8) to eliminate *f*_g_ from equation (14), one obtains
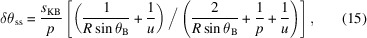
which is identical to the contribution of a finite source to the angular resolution of a concave crystal (Phizackerley *et al.*, 1983 ▸) but with terms resulting from divergence and crystal curvature summed instead of subtracted, as was also the case for the energy bandwidth and resolution due to the finite detector pixel size.

#### 3.2.3. Intrinsic resolution of the analyzer crystal

**Figure 9:**
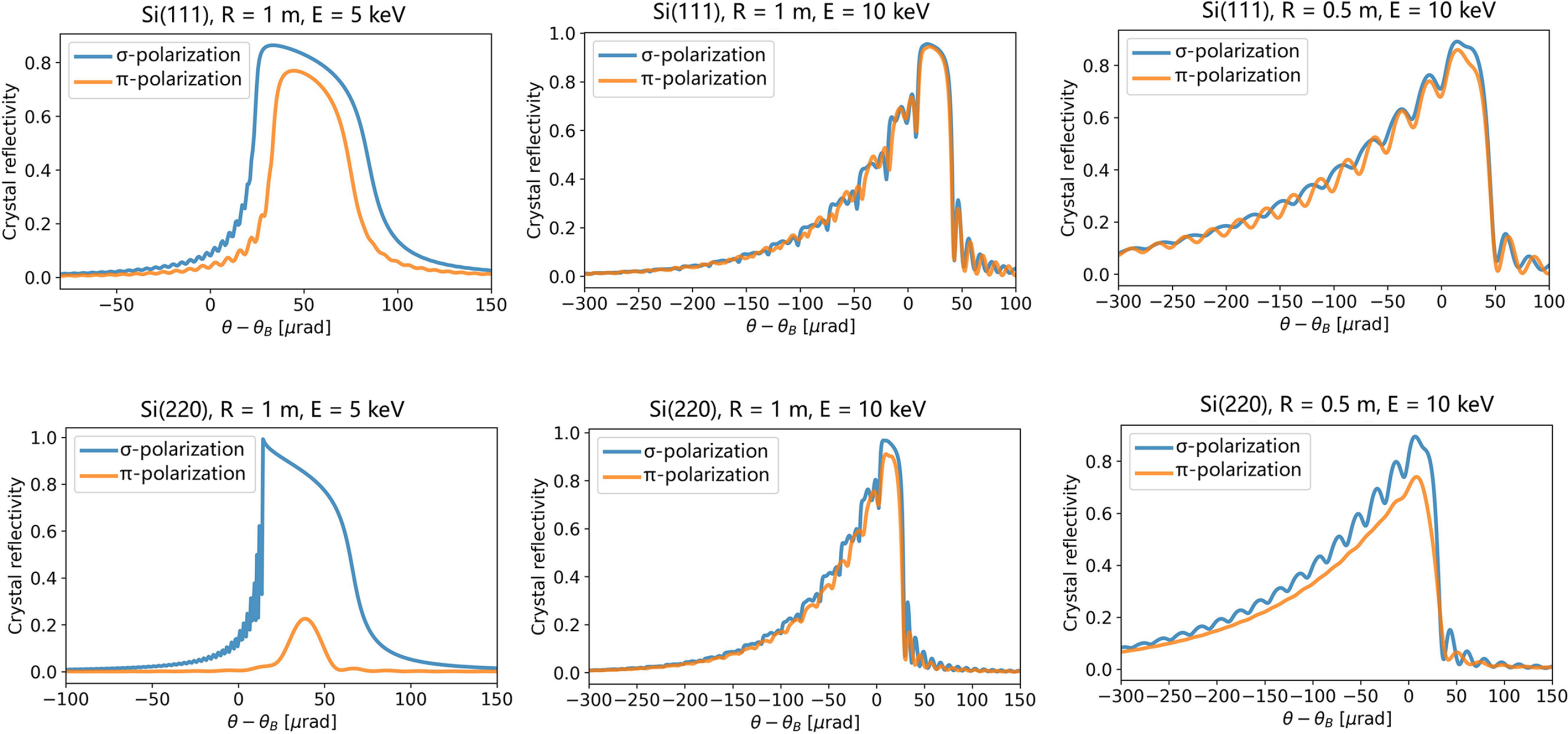
Crystal reflectivity profiles simulated by the *XOPPYLIB* package. Blue and orange lines show σ-polarization and π-polarization, respectively. For the geometry of the instrument presented herein, where X-rays diffract from the polychromator in the horizontal plane, π-polarization should be considered.

The crystal’s intrinsic angular resolution (δθ_int_) is equal to the FWHM of the crystal’s diffraction (or reflectivity) profiles. For bent crystals in Bragg geometries, this profile has contributions from the Darwin width of the crystal reflection and the strain introduced by bending and can be modeled using the multilamellar method (Sanchez del Rio *et al.*, 2015 ▸; Caciuffo *et al.*, 1990 ▸; Erola *et al.*, 1990 ▸). This method is included in the *XOPPYLIB* package, which we used for the simulations presented herein. Example simulations for cylindrically bent Si(111) and Si(220) crystals with a radius of 1.0 m at 5 and 10 keV and a radius of 0.5 m at 10 keV are provided in Fig. 9 ▸.

### 3.3. Selection of DXAS instrument geometry

In order to obtain optimal X-ray absorption spectra, a DXAS instrument that provides a broad energy spread and minimal energy resolution is desired. The maximum energy spread achievable is limited by the polychromatic spectrum incident on the sample, which in this case is determined by the undulator source and the multilayer monochromator. In principle, the undulator bandwidth can be increased by tapering its magnets. We, therefore, consider the multilayer bandwidth to be the limiting factor for simplicity. The minimum energy resolution achievable is set by the core-hole lifetime broadening of the absorption edge under study. The optimal instrument would therefore match the crystal’s energy range to that of ML 48 while maintaining the total energy resolution below the *K*-shell core-hole-lifetime broadening, which is smaller than that of the corresponding *L*-shell. The energy spread and the spectrometer’s resolution are influenced by several parameters, as discussed above: the sample-to-crystal distance (*p*), the crystal-to-detector distance (*u*), the crystal’s bending radius (*R*), the Bragg angle (θ_B_), the Miller indices (*h*, *k*, *l*) of the crystal reflection, the divergence (ω), and the pixel-array detector’s pixel size (*s*_pix_). Some of these parameters have fixed values, including the detector’s pixel size (*s*_p_) and the divergence (ω), which is determined by the KB mirrors. The mirrors have fixed elliptical shapes and are, therefore, not tunable. In the subsequent discussion, we will detail how the values for *p*, *u*, *R*, θ_B_, and the Miller indices can be determined.

#### 3.3.1. Sample-to-crystal (*p*) and crystal-to-detector (*u*) distances

A larger sample-to-crystal distance, *p*, will increase the total energy spread of the spectrometer, according to equation (5), and minimize the energy resolution broadening contributed from the KB focus size, according to equation (15). A bigger crystal-to-detector distance, *u*, will reduce the energy resolution broadening effects from both the detector pixel size and the KB focal size, as seen in equations (10) and (14). Therefore, relatively large distances between the sample, analyzer, and detector are desirable. A fixed value of 1.5 m is selected for *p*, as values beyond this are impractical to implement due to the spatial constraints imposed by other instrumentation within the endstation hutch. The distance *u* is chosen to be the maximum value at which the area detector can capture the entire Bragg beam, but it is limited to a maximum of 2.5 m by the spatial constraints of the experimental hutch.

#### 3.3.2. Bragg angle θ_B_, Miller indices (*h*, *k*, *l*), and bending radius *R*

**Figure 10:**
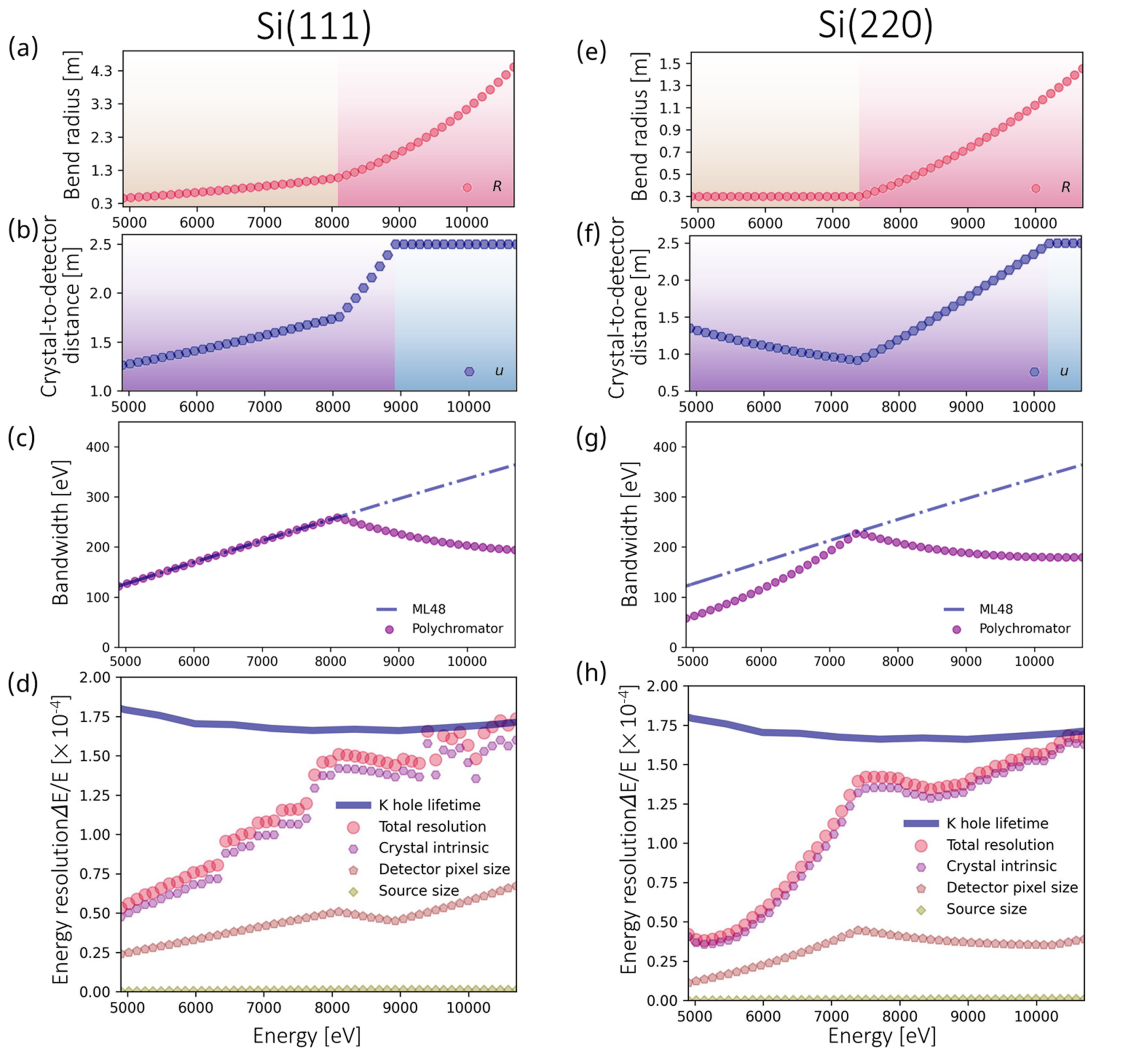
Operating parameters for the DXAS instrument with focus-to-crystal distance (*p*) set to 1.5 m and X-ray divergence (ω) fixed at 1.2 mrad by the KB mirrors for (*a*–*d*) Si(111) and (*e*–*h*) Si(220) analyzer crystals. (*a*, *e*) The bending radii (*R*) of the polychromators at different energies. In the orange region, the *R* values are determined by matching the energy spread of the polychromator to that of ML 48 where possible without reducing the radius below a minimum value of 0.3 m. In the red region, the *R* values are chosen so that the total energy resolutions [shown in (*d*) and (*h*)] do not exceed the *K*-shell core-hole lifetime broadening. (*b*, *f*) The crystal-to-detector distances (*u*) at different energies. In the purple region, the *u* values are determined by constraining the Bragg beam size to 28 mm so that the pixel array detector can capture the entire Bragg beam. In the blue region, *u* is fixed at 2.5 m so as not to exceed the hutch’s spatial constraints. (*c*, *g*) The energy bandwidths of the DXAS polychromators in comparison with the ML 48 bandwidth. (*d*, *h*) The corresponding energy resolutions of the DXAS instrument in the operating energy ranges of ML 48 (4.9 keV to 10.7 keV) in comparison with the *K*-shell core-hole lifetime broadening, which is obtained from Campbell & Papp (2001 ▸).

The energy resolution of the final absorption spectrum is dominated by the intrinsic energy resolution of the analyzer crystal, which is affected by the strain introduced by crystal bending, the Bragg angle, and the Miller indices of the crystal reflection being used. As the curvature is increased, the strain increases, which increases the energy resolution. This is illustrated in Fig. 9 ▸, which shows reflectivity profiles simulated by the *XOPPYLIB* package for bent Bragg silicon crystals. In addition to bending, reflections for which the sum of the Miller indices is lower also provide broadened energy resolution, as does lower Bragg angle, as indicated by equation (7). Therefore, to maintain the energy resolution below the *K*-edge core-hole-lifetime broadening, a large bending radius can be selected at the highest energies where the Bragg angle is the smallest. As lower energies are accessed by increasing the Bragg angle for a given reflection, the bending radius can be reduced in order to increase the energy bandwidth, as indicated by equation (5). In doing so, the loss of energy resolution resulting from the reduced bending radius is offset by the increase in Bragg angle. The minimum bending radius was restricted to a value of 0.3 m in order to limit the maximum stress applied to the crystal. The energy resolution and bandwidth obtained using the strategy described above are shown for Si (111) and (220) reflections in Fig. 10 ▸. These two reflections are commonly used in X-ray monochromators and analyzers. Panels (*a*), (*b*), (*e*), and (*f*) of Fig. 10 ▸ show the selected bending radii and analyzer-to-detector distances for the two crystal reflections as a function of energy, and panels (*d*) and (*h*) show the effect on energy resolution. At low energies, the bending radius for Si (111) can be selected to match the energy bandwidth of the analyzer to that of the multilayer monochromator, as shown in panel (*c*), and the energy resolution remains well below the core-hole lifetime. At higher energies, the needed use of a large radius to maintain energy resolution below the core-hole lifetime broadening results in an energy bandwidth below that of ML 48 for both Si (111) and (220) reflections. For Si (220), the energy bandwidth remains below that of ML 48 for most of the energy range due to the desire to minimize stress in the crystal by limiting the minimum bend radius. Consequently, a Si (111) polychromator is better suited in the lower energy range. Nevertheless, comparing panels (*c*) and (*g*) of Fig. 10 ▸, one observes that similar bandwidths are provided by the Si (111) and Si (220) reflections, and the energy ranges provided by both reflections are sufficient for the collection of XANES spectra.

## 4. Data analysis and energy calibration

**Figure 11:**
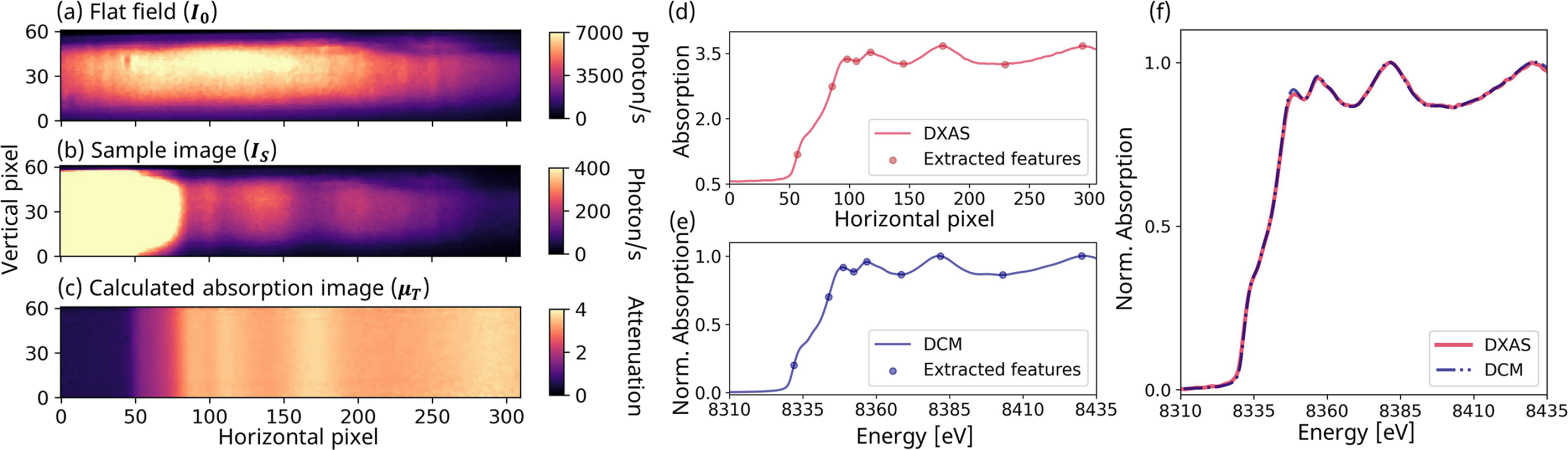
X-ray intensity images recorded by the pixel-array detector (pixel size of 55 µm) (*a*) without any sample, essentially a flatfield, denoted as ***I***_0_, and (*b*) with a nickel-foil sample, denoted as ***I***_S_. The images shown here are cropped from the full 500 × 500 pixel field-of-view of the detector for better visualization. (*c*) The corresponding absorption image, denoted as **μ**_T_, calculated pixel-wise by Lambert–Beer’s law. A median filter with a kernel size of 5 was applied to remove impulse noise. (*d*) Generated XANES spectrum as a function of pixel number from the absorption image. (*e*) A ‘standard’ spectrum measured at a scanning DCM-based beamline (APS 20-BM) with the energy axis calibrated. In (*d*) and (*e*) several corresponding feature points are selected, which are used for energy calibration of the DXAS spectrum. A polynomial fit using the feature points yields an energy calibration function. (*f*) The final DXAS spectrum after application of the energy calibration function and the standard DCM spectrum.

To obtain an absorption spectrum from the raw images acquired by the pixel array detector, several image processing steps are required. First, an absorption image is calculated pixel-wise using Lambert–Beer’s law, which is given by
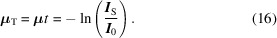
Here **μ**_T_ is the total absorption, which is the absorption coefficient (**μ**) multiplied by the sample thickness (*t*), ***I***_S_ is the image acquired from the sample, and ***I***_0_ is a flatfield image acquired without the sample in place. The ***I***_0_, ***I***_S_, and **μ**_T_ images of a nickel foil are shown in Figs. 11 ▸(*a*), 11(*b*), and 11(*c*), respectively. The images were collected prior to the APS upgrade for an exposure time of 5 s using an Si(111) polychromator with a bending radius of 2 m and with the distances *p* and *u* set to 1.2 m and 2.5 m, respectively. These instrument parameters were chosen to provide an energy resolution of 1.25 eV at the Ni *K*-edge, which is better than the Ni *K*-edge core-hole-lifetime broadening of 1.39 eV (Campbell & Papp, 2001 ▸), and were implemented before the parameters in Fig. 10 ▸, which maintain a more compact arrangement of components, were determined. A 2D median filter with kernel size of 5 was applied to the **μ**_T_ image to suppress impulse noise, commonly known as ‘pepper and salt’ noise, which appears as isolated bright and dark pixels (Huang *et al.*, 1979 ▸). Such filters are commonly used in image processing.

Often, a slight tilt in the intensity modulation lines is observed, which may result from miscut, misalignment, or bending distortions of the crystal wafer. We corrected this by simply rotating the image so that the absorption lines are oriented vertically. When dealing with curved features, a non-linear warping of the images can be applied to make corrections (Huang *et al.*, 2021 ▸). Averaging all values along the vertical direction generates a preliminary XANES spectrum as a function of horizontal pixel number, as shown in Fig. 11 ▸(*d*).

In order to calibrate the energy scale of the detector images, we employ a standard absorption spectrum with known energy axis. In this case, we use a XANES spectrum collected at a scanning DCM-based XAFS beamline from the same nickel foil. The same feature points, including local maxima and edge points, were automatically selected for these two spectra, shown in panels of Figs. 11 ▸(*d*) and 11(*e*), using built-in Python functions from the *Scipy* package (Virtanen *et al.*, 2020 ▸). The edge points were defined as the points on the rising edge where the absorption was 20% and 80% of the difference between the absorption below the rising edge and the maximum of the first XANES peak. By applying second- or third-degree polynomial fitting, we generated a fitting function that aligns the feature points of the DXAS spectrum with those of the DCM spectrum. This function was then applied to all data points of the DXAS spectrum, thereby calibrating every pixel position to its final X-ray energy.

Fig. 11 ▸(*f*) compares the energy-calibrated DXAS spectrum with the DCM spectrum. The two spectra closely resemble each other except for subtle differences near the white line, possibly due to distortions in the crystal induced by local defects or an inhomogeneous heat load. Nevertheless, this indicates the robustness of the calibration method and demonstrates the spectrometer’s ability to measure high-quality spectra in which all spectral features are well resolved.

## 5. Application to a lithium-ion battery cathode material

To demonstrate the DXAS instrument capability, we measured *ex situ* XANES of lithium-transition-metal-oxide battery cathodes. Lithium-ion batteries (LIB) are crucial for modern technology, and the selection of a cathode material is among the most critical aspects, with transition-metal oxides typically employed. XAFS proves invaluable in the analysis of these compounds primarily because, during the processes of lithiation and delithiation, the oxidation states of the transition metals in these active materials change (Nowack *et al.*, 2016 ▸).

Among transition-metal oxides, lithium- and manganese-rich compounds are good candidates for future lithium-ion battery cathodes with limited cobalt and nickel (Gutierrez *et al.*, 2023 ▸). As a cathode, the transition-metal oxide composition 0.3Li_2_MnO_3_·0.7LiNi_0.5_Mn_0.5_O_2_ has significant nickel redox behavior, with limited manganese redox, and is, therefore, an interesting candidate for initial studies. Dispersive XANES was collected *ex situ* from Li_1.13_Ni_0.30_Mn_0.57_O_2_ (0.3Li_2_MnO_3_·0.7LiMn_0.5_Ni_0.5_O_2_) at multiple states of charge: Pristine (uncycled), 3.5 V, 4.3 V and 4.6 V. The cathode material was prepared in a process equivalent to that described by Chen, Gutierrez *et al.* (2021 ▸). After being deposited onto a 20 µm-thick aluminium current collector, the Li_1.13_Ni_0.30_Mn_0.57_O_2_ electrodes (14 mm diameter) were combined with a polyolefin separator and a Li-metal anode (15.6 mm diameter). For cycling, electrodes were assembled into 2032-format coin cells with 26 µL of a 1.2 *M* LiPF_6_ electrolyte in a 3:7 weight ratio mixture of ethyl carbonate and ethyl-methyl carbonate (Tomitama A49). Cells were cycled using a Maccor Series 4000 battery test system. After a three-hour rest, the cells underwent three cycles of charging at 20 mA g^−1^ to 4.4 V and discharging at 20 mA g^−1^ to 2.5 V, followed by two cycles of charging at 10 mA g^−1^ to 4.7 V and discharging at 10 mA g^−1^ to 2.5 V. The cells were held at 2.5 V until the current dropped below 5 mA g^−1^, and finally charged at 20 mA g^−1^ to 3.5 V, 4.3 V, or 4.6 V, depending on the sample, with a constant-voltage hold. After cycling, the cells were disassembled. Both cycled and pristine Li_1.13_Ni_0.30_Mn_0.57_O_2_ cathodes were encased in Kapton tape to ensure chemical stability before being transported to the beamline. The DXAS measurements were performed prior to the APS upgrade under ambient conditions, specifically at room temperature and in an open-air environment, using a Si (111) polychromator with a bending radius of 2 m and with the distances *p* and *u* set to 1.8 m and 1.6 m, respectively. These spectra were collected prior to the final determination of instrument operating parameters and thus differ from the values indicated in Section 3.3. Nevertheless, these parameters were also chosen to provide energy resolution and bandwidth sufficient for the collection of XANES spectra. Using the method provided in Section 3.2, a total energy resolution of 0.91 eV at the Mn *K*-edge is calculated for these parameters, which is better than the Mn *K*-edge core-hole-lifetime broadening of 1.11 eV (Campbell & Papp, 2001 ▸). The exposure time for the pristine sample and the samples charged at 4.6 V and 3.5 V was 120 s, with a total of 2 × 10^7^ to 7 × 10^7^ photons counted by the detector, *i.e.* 2 × 10^5^ to 6 × 10^5^ photons s^−1^. The exposure time for the sample charged at 4.3 V was 240 s, with a total of 3 × 10^8^ photons counted,*i.e.* 1.2 × 10^6^ photons s^−1^. The exposure time for the flatfield image was 5 s, with a total of 1.7 × 10^9^ photons counted,*i.e.* 3.3 × 10^8^ photons s^−1^. A median filter with a kernel size of 5 was applied to the processed absorption images to remove impulse noise.

**Figure 12:**
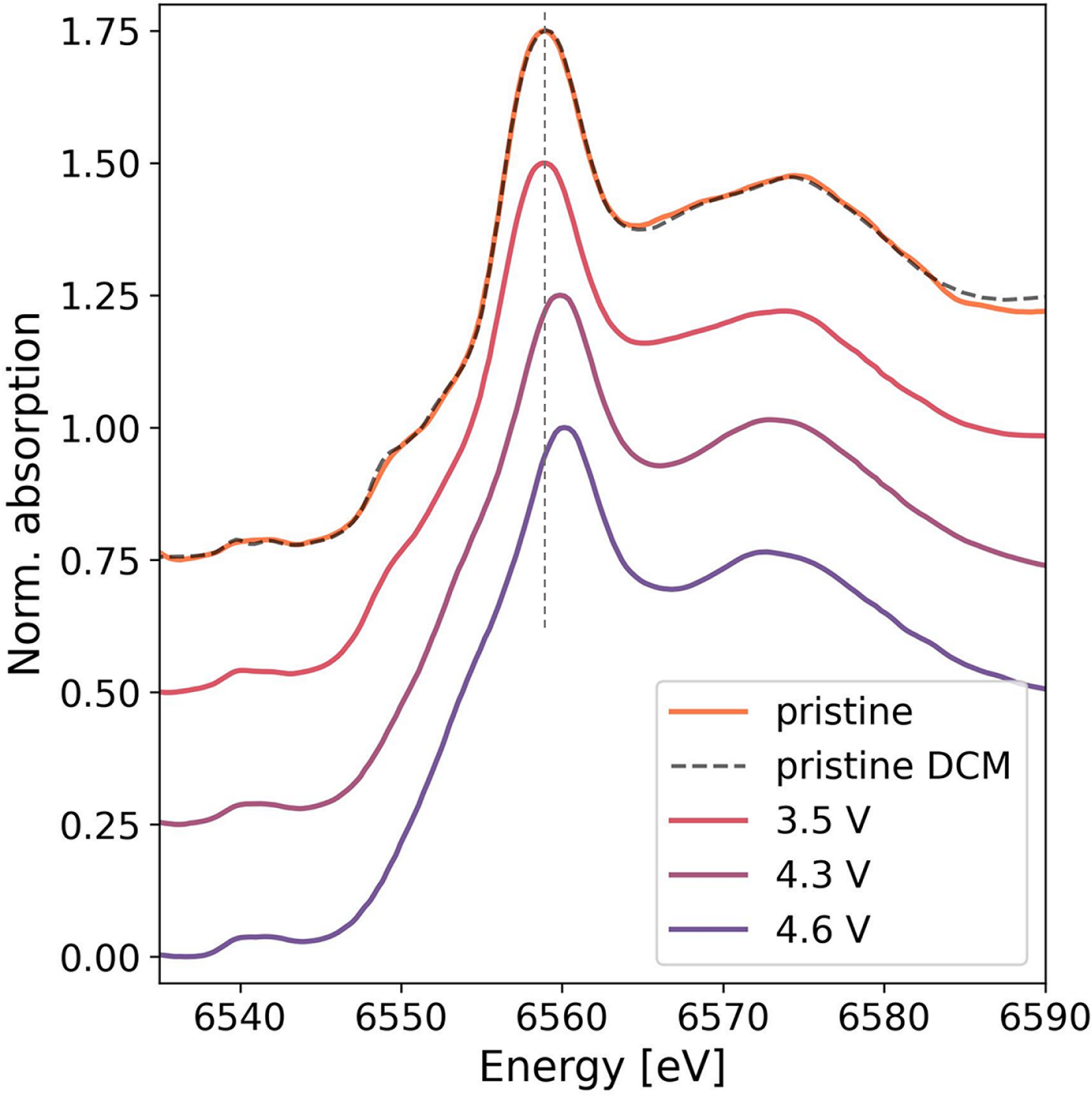
Mn *K*-edge XANES spectra of Li_1.13_Ni_0.30_Mn_0.57_O_2_ under different states of charge measured using the DXAS spectrometer and of the pristine material measured using both the DXAS spectrometer and a conventional DCM beamline. The spectra are offset vertically to facilitate comparison.

Fig. 12 ▸ shows XANES spectra of the Li_1.13_Ni_0.30_Mn_0.57_O_2_ at different charge potentials, *i.e.* 3.5 V, 4.3 V, and 4.6 V, and in a pristine state. With increasing voltage, the spectral features shift to higher energies, indicating an increase in the Mn oxidation state, which is consistent with the electrochemical extraction of Li that occurs during charging. Quantitative analysis of the oxidation state typically requires comparison with reference materials of known oxidation state and was therefore not carried out for the present proof-of-principle measurements. For comparison, Mn *K*-edge XANES of the pristine material measured on a conventional DCM beamline is also shown. The measurement at the DCM beamline was conducted at a different time using a newly prepared pristine sample. XANES collected with the DXAS spectrometer and the DCM beamline show spectral features with the same relative intensities at the same energy positions. Some subtle differences in intensity can be observed, particularly at the high energy end of the spectra, which may be attributed to differences in the spectral backgrounds resulting from variation in sample preparation and X-ray beam spot size.

## 6. Sample and performance consideration

A significant feature of the DXAS instrument presented here is the use of separate optics for focusing and dispersion of the X-ray beam, which results in some characteristics differing from those of instruments that use a curved crystal to simultaneously perform both functions. A Bragg crystal concavely bent to a perfect ellipse will focus all diffracted rays to the same point. Imperfections in the shape of the crystal result in variation of the focal distance, and X-rays diffracted from different regions will therefore not all intersect at the same point in space. This leads to rays of different energies, which are diffracted from different positions on the crystal, intersecting the sample at different points, which in turn gives rise to artifacts in spectra collected from samples that are not homogeneous. In order to minimize such chromatic aberrations, stringent bending requirements can be imposed and may require adjustment of the crystal at each absorption edge. Grazing-incidence optics are achromatic, and therefore the use of KB mirrors to focus avoids this issue. Furthermore, use of a Bragg crystal only for dispersion means bending requirements can be relaxed, which simplifies polychromator design and operation.

Using ultraprecise fabrication methods, a high degree of perfection can be achieved in KB-mirror shapes. Nano­focusing can be realized with such low-figure-error KB mirrors. If combined with the instrument geometry described here, this can enable high-spatial-resolution XANES mapping to examine nanoscale variation in the physical properties of materials, such as oxidation state.

Without needing for the Bragg crystal polychromator to focus the beam, it can be convexly bent, which increases the angular dispersion of the X-rays that it diffracts. This can be seen from equation (1), which shows that the contributions from crystal curvature and divergence of the incoming beam are summed. On the other hand, for a concave crystal, ω is subtracted from *L*/*R*, resulting in less dispersion. The increased dispersion of the convex crystal improves the energy resolution of a pixel array detector located a fixed distance from the polychromator.

Because the polychromator is located downstream of the sample, the sample receives the full focused flux of the multilayer monochromator, which, as shown in Table 4 ▸, carries a power of 0.1–0.9 W. The subsequent interaction with the polychromator significantly reduces the flux received by the detector because of the narrow band pass of the crystal, which diffracts each incident energy from only specific limited regions. This results in many of the photons that interacted with the sample going unused. With the polychromator placed upstream of the sample, conventional DXAS avoids this issue and provides the sample with a lower flux where all transmitted photons contribute to the absorption spectrum within the detector efficiency. This will achieve spectra with a similar signal-to-noise ratio while delivering less flux to the sample. Because the ASB DXAS instrument delivers more flux to the sample, one must carefully consider the potential impact of radiation damage on the sample and make accommodations to reduce beam damage to the extent possible, such as by using a cryostat or, for homogeneous samples, by moving to different regions of the sample with repeated measurements.

The energy range of the ASB DXAS instrument is determined by the bandwidth of the multilayer monochromator, which is ∼3% in the primary operating range. This bandwidth roughly matches that of APS undulator A (U33). As discussed in Section 2.1, the bandwidth of the APS-U undulator (U28) is reduced, and tapering is required to match that of ML 48, introducing an additional tradeoff between flux and bandwidth. These constraints also restrict the wavevector range that would be obtained in an EXAFS spectrum and limit the instrument to the XANES regime. Potentially, EXAFS spectra could be attained by combining multiple spectra obtained over different energy ranges, which requires synchronous adjustment of the multilayers and the polychromator.

The calculated flux of the DXAS instrument is 10^11^ to 10^12^ photons s^−1^ at the detector (Table 4 ▸), in the absence of attenuation from decoherers, helium paths, attenuation filters, and samples. This yields about 10^7^ to 10^8^ photons s^−1^ pixel^−1^ over the area of a typical image, which is approximately 500 × 60 pixels. The LAMBDA 250 K pixel array detector can collect a maximum of ∼8 ×10^5^ photons s^−1^ pixel^−1^, which is one to two orders of magnitude below that which the instrument can achieve in the absence of attenuation. Therefore, the maximum count rate of the detector determines the ultimate exposure time required. The experimental measurements presented herein show that a total count rate of about 10^3^ photons pixel^−1^ on the detector provide sufficient data quality. In principle, acquisition of a XANES spectrum can therefore be achieved in a few milliseconds when operating at the maximum count rate of the detector, which is attainable in the absence of attenuation filters and with minimal air along the beam path.

The fast acquisition time that we anticipate can be achieved with this instrument will increase the speed with which one can collect XANES spectra by three orders of magnitude at the ASB, where the Si (111) DCM is not of the oscillating or air-bearing type. This enhanced time resolution will enable new scientific research opportunities at the ASB. In particular, the kinetics of chemical transformations and mechanisms of catalytic reactions can be examined *in situ* and *in operando* with high temporal resolution. The DXAS capability can also be valuable for pump–probe spectroscopy to examine dynamic processes at nanosecond timescales by enabling collection of a full spectrum with each probing event.

For many experiments, fluorescence detection, which requires measuring the intensity of emitted photons of fixed energy across the absorption edge, may be desired. When using the DXAS technique, all energies of the absorption edge are incident on the sample simultaneously, and thus, fluorescence detection cannot be used. Conventional DXAS instruments overcome this limitation by scanning a slit located between the polychromator and sample through the incident beam (Pascarelli *et al.*, 1999 ▸). Fluorescence is detected as the slit moves through the X-ray beam, selecting narrow energy ranges defined by the slit width as it moves. This method for collecting fluorescence XAS would not be possible with the ASB DXAS instrument because the polychromator is placed after the sample. However, fluorescence XAS is readily achieved at the ASB by using the Si (111) crystal set of the DCM. Because of the many techniques and XAS detection schemes enabled by the Si (111) DCM, as enumerated in Section 2, we anticipate that it will remain the primary mode of operation for the ASB. As noted above, the DXAS instrument is optimal for time-resolved studies of samples suitable for transmission detection in which radiation damage can be mitigated or is not a concern.

When conducting experiments with the DXAS instrument, the modular design, where polychromator and pixel array detector are integrated with existing beamline focusing optics and sample stages, makes it easy to implement and allows the beamline to readily transition between different experimental setups. Furthermore, due to its modularity and simplicity, this instrument or instruments of the same geometry can be straightforwardly implemented at other beamlines with polychromatic sources.

## 7. Conclusion and outlook

We have designed and demonstrated an instrument for dispersive XANES spectroscopy that uses a convex curved Bragg crystal as the spectrometer to disperse X-rays transmitted through a sample placed at the focal position of a KB mirror pair onto a high-quantum-efficiency (>90%) pixel-array detector. Spectra collected from standard reference materials and metal-oxide laminates display good spectral quality with energy resolution below the *K*-shell core-hole-lifetime broadening. Due to bandwidth limitations imposed by the multilayer monochromators, EXAFS spectra would be limited in wavevector range or require multiple measurements over different energy ranges.

The operational X-ray energy range of ML 48 is between 4.9 keV and 10.7 keV. Over the operating energy range of ML 24, which is between 9.8 keV and 21.5 keV, the intrinsic energy resolution of the Bragg polychromator significantly increases. Thus, transitioning the instrument to a Laue geometry can be considered a potential route to operating above the present energy range.

A significant feature of our dispersive instrument is that X-ray focusing and energy dispersion are accomplished using two independent optical components. In contrast, many dispersive beamlines have polychromators that simultaneously perform both functions, leading to considerable challenges in polychromator design. For instance, precise control of slope errors and bending shape (typically elliptical) is required to achieve a small X-ray focal spot, and adjustments are necessary to accommodate different energy ranges for various absorption edge measurements.

A drawback of positioning the polychromator downstream of the sample is an increase in the photon flux on the sample, which can induce beam damage. However, the proposed setup also offers several advantages, such as a reduced need for complex cooling systems due to the lower X-ray flux on the polychromator and a modular endstation setup that increases flexibility and practicality in the instrument’s arrangement. Therefore, for beamlines equipped with a polychromatic incident beam, especially those not originally designed for XAS, this approach can serve as a straight­forward add-on technique that expands the beamline’s capabilities.

The experimental measurements presented herein show that a total count rate of about 10^7^ photons s^−1^ on the detector provides sufficient data quality. To enhance future experiments, a set of longer KB mirrors is being developed, which will achieve submicrometre focusing and increase the X-ray flux by a factor of four due to greater beam acceptance. Additionally, the removal of attenuation filters and air from the beam path, combined with the recently completed upgrade of the APS to a fourth-generation light source, is expected to further increase incident X-ray flux. Therefore, we anticipate that, in the future, these advancements will reduce the exposure times required by at least three orders of magnitude. As a result, the exposure time for a sample with an optimal concentration (*e.g.* approximately 1.5 absorption length) is expected to decrease to just a few milliseconds.

A conventional Si (111) DCM is typically employed for XAFS at the ASB. The DXAS instrument offers a complementary fast acquisition scheme. This fast single-shot method for collecting XANES provides new opportunities for advancing time-resolved and pump-and-probe spectroscopy at 25-ID. Furthermore, when combined with fly scanning of the sample stages, we expect to achieve 2D XANES mapping of more than 100 × 100 pixels within a minute. In addition, a beryllium lens (Heald & Dufresne, 2018 ▸) installed downstream of the KB mirrors will facilitate the enlargement of the KB focal spot size and thereby enable flexible control of experimental conditions. This can allow more efficient (*e.g.* adjustment of spatial resolution based on region-of-interest) and more intelligent (*e.g.* combined with machine-learning algorithms) data collection schemes.

## Figures and Tables

**Table 1:** Comparison of APS and APS-U electron beam parameters

	APS-U brightness mode	APS-U timing mode	APS
Electron beam energy (GeV)	6	6	7
Electron beam current (mA)	200	200	100
Number of electron bunches	324	48	24
Electron horizontal size, σ_*x*_ (µm)	14.8	12.9	275
Electron vertical size, σ_*y*_ (µm)	3.7	8.7	10.3
Electron horizontal divergence (µrad)	2.8	2.5	11.3
Electron vertical divergence (µrad)	1.5	3.6	3.6

**Table 2:** Summary of the DXAS instrument parameters at the ASB

Source	APS: canted U33 (undulator A), 33 mm period, number of periods = 62
	APS-U: canted U28 undulator, 28 mm period, number of periods = 75
Deflecting mirrors	Uncoated Si, Rh-coated, and Pt-coated stripes
KB mirrors	Rh coated (<20 keV)
	Vertical mirror: 100 mm long
	Horizontal mirror: 60 mm long, increased to 100 mm with APS-U
Sample stage	Aerotech nanopositioning stages ANT130L&LZ
Detector	LAMBDA 250 K, 55 µm × 55 µm pixel size, 28 mm × 28 mm field-of-view

**Table 3:** Specifications of Mo/B_4_C multilayers used in the ASB monochromator

Multilayer	ML 48 (*d* = 48 Å)	ML 24 (*d* = 24 Å)
Dimensions (mm) (L × W)	∼90 × 34	∼90 × 34
Multilayer coating	Mo (12 Å) + B_4_C (36 Å)	Mo (10 Å) + B_4_C (14 Å)
Number of bilayers	80	300
Angle range	0.7–1.5°	0.7–1.5°
Energy range	4.9–10.7 keV	9.8–21.5 keV
Energy bandwidth	∼3%	∼1%

**Table 4:** Flux and power output from critical beamline and instrument components with the DXAS instrument operated at 8 keV Calculations were performed for the APS and the APS-U using a white-beam-slit size that matches the acceptance of the DXAS instrument, which is limited by the lengths of the KB mirrors, given in Table 2 ▸. The flux for all components was calculated using only the first harmonic of the undulator. The power output of the undulator was calculated using all harmonics, and that of all other components was calculated using only the first harmonic, as all harmonics other than the first have energies above the 12.6 keV cutoff energy of the Si regions of the high-heat-load mirrors. For APS-U, we consider tapering the undulator by 0.5 keV, as discussed in the previous section, and bending the second high-heat-load mirror to collimate the beam, which is the planned standard operating configuration of the mirror. For APS, we consider the undulator without tapering and flat high-heat-load mirrors, which is the configuration used for experiments discussed herein. The power of undulator harmonics other than the first were calculated without tapering for both the APS and APS-U.

	APS (configuration used in this work)		APS-U (future DXAS instrument configuration)	
Component	Flux output (photons s^−1^)	Power output (mW)	Flux output (photons s^−1^)	Power output (mW)
Undulator and slit	1.9 × 10^14^	360	1.8 × 10^15^	4200
High-heat-load mirrors	1.7 × 10^14^	220	1.7 × 10^15^	2110
Multilayers (ML 48)	8.9 × 10^13^	110	7.4 × 10^14^	945
KB mirrors (Rh)	8.3 × 10^13^	106	6.7 × 10^14^	877
Si (111) polychromator	7.0 × 10^11^	0.9	5.2 × 10^12^	6.7

**Table 5:** Flux and power output calculated for APS-U undulator with different sizes of the white beam slits located 27 m from the source

White beam slit size (at 27 m) (horizontal × vertical)	Total power (all undulator harmonics)
2 mm × 2 mm	365.8 W
1 mm × 1 mm	102.9 W
500 µm × 500 µm	26.5 W
264 µm × 150 µm (DXAS APS-U)	4.2 W
